# A robotic treadmill system to mimic overground walking training with body weight support

**DOI:** 10.3389/fnbot.2023.1089377

**Published:** 2023-06-09

**Authors:** Jongbum Kim, Seunghue Oh, Yongjin Jo, James Hyungsup Moon, Jonghyun Kim

**Affiliations:** ^1^Department of Robotics and Mechatronics Engineering, Daegu Gyeongbuk Institute of Science and Technology (DGIST), Daegu, Republic of Korea; ^2^Department of Physical Therapy, Uiduk University, Gyeongju-si, Republic of Korea; ^3^Department of Mechanical Engineering, Sungkyunkwan University, Suwon-si, Republic of Korea

**Keywords:** body weight support, overground walking training, treadmill, wire mechanism, gait rehabilitation

## Abstract

**Introduction:**

Body weight support overground walking training (BWSOWT) is widely used in gait rehabilitation. However, existing systems require large workspace, complex structure, and substantial installation cost for the actuator, which make those systems inappropriate for the clinical environment. For wide clinical use, the proposed system is based on a self-paced treadmill, and uses an optimized body weight support with frame-based two-wire mechanism.

**Method:**

The Interactive treadmill was used to mimic overground walking. We opted the conventional DC motors to partially unload the body weight and modified pelvic type harness to allow natural pelvic motion. The performance of the proposed system on the measurement of anterior/posterior position, force control, and pelvic motion was evaluated with 8 healthy subjects during walking training.

**Results:**

We verified that the proposed system was the cost/space-effective and showed the more accurate anterior/posterior position than motion sensor, comparable force control performance, and natural pelvic motion.

**Discussion:**

The proposed system is cost/space effective, and able to mimic overground walking training with body weight support. In future work, we will improve the force control performance and optimize the training protocol for wide clinical use.

## 1. Introduction

Gait function recovery is one of the most important goals of stroke and spinal cord injury patients (Guralnik et al., 1993). The body weight support system (BWS), which supports the partial weight of a patient, helps maintain the balance of the patient (Pina et al., 2018) and can control gait training intensity with various unloading forces (Vaughan, 1996). Hence, body weight support treadmill training is widely used in gait rehabilitation. Recently, body weight support overground walking training (BWSOWT) has been applied to improve the patient's gait function (Wyss et al., 2014). This is because overground walking (OW) is promising, in that it is the ultimate goal of gait rehabilitation, and promotes voluntary walking (Riley et al., 2007; Yen et al., 2012; Oh et al., 2018).

There have been several prior studies in developing BWSOWT systems, such as Zero-G, Vector, and FLOAT. They provided unloading force using wires from the ceiling during OW, and allowed various movements, such as turning, curved movement, or lateral movement on overground (Hidler et al., 2011; Vallery et al., 2013), but have limitations (Vallery et al., 2008; Plooij et al., 2018) to being widely used in a clinical environment of large required space and high cost. These limitations are due to their large workspace, complex structure, and installation cost at the ceiling. Another approach for providing BWSOWT is the mobile robot with attached harness that can unload the patients' weight and assist walking, such as KineAssist (Peshkin et al., 2005) and Andago (Hocoma, Switzerland), and they could enable curved movement like OW. However, KineAssist showed limitations of the walker finding it hard to move fast due to their heavy structure (Vallery et al., 2013), and the restriction of mobility caused by their size (Seo and Lee, 2009; Mun et al., 2015) and delay in responsiveness (Hidler et al., 2011). Those limitations were improved by Andago, but still required the large workspace for BWSOWT (van Hedel et al., 2021).

A promising approach to overcome the limitations of the existing BWSOWT systems is to mimic OW on a treadmill that includes a simple (optimized) BWS. The self-paced treadmills, which have been developed to enable the user to adjust the walking speed based on a treadmill belt speed controller (Minetti et al., 2003; Fung et al., 2006; Souman et al., 2010; Feasel et al., 2011; Yoon et al., 2012; Kim et al., 2015), could be used for the approach. Since it easily allows the user to accelerate and decelerate on the treadmill, walking on the treadmill becomes physically similar to OW, with taking advantages of the treadmill, of small required space and affordable cost (Riley et al., 2007; Scherer, 2007). Furthermore, our study has recently shown that the use of a self-paced treadmill that we developed, the interactive treadmill (ITM), can increase the user's attention to gait, which could facilitate brain plasticity (Oh et al., 2018).

However, despite those advantages of the self-paced treadmill, there were few studies to develop and implement the BWSOWT using a self-paced treadmill, 3-wire (Sabetian and Hollerbach, 2017) and KineAssist-MX (Woodway, USA). However, they had some components which could be burdensome for natural gait, such as mechanical tether (3-wire) and pelvic support arm (KineAssist-MX). Moreover, 3-wire still required large space due to both its custom treadmill used and the actuators installed at ceiling. Note that KineAssist-MX could suffer from anomalous (inertial) force, which is different from OW, due to its significant belt acceleration for implementing self-paced treadmill while our ITM did not (will be mentioned in Section 2.1) (Kim et al., 2014).

The goal of this paper is to develop a novel treadmill-based gait training system to overcome the limitations of the existing BWSOWT systems, with the following characteristics: cost-effective and non-oversized for better clinical use, simple and unburdensome to mimic natural OW. For those, the proposed system is based on a self-paced treadmill, ITM, because (1) it is an optimal version implemented without expensive devices, such as large treadmill, motion capture system, or force plate (Souman et al., 2010; Yoon et al., 2012), and (2), and its similarity to overground walking has been verified in terms of both physical and mental activities training (Oh et al., 2018). As to BWS, a customized BWS for ITM with a novel 2-wire mechanism was developed to achieve optimal size and cost for better clinical use, and a novel harness system was proposed to allow pelvic motion for mimicking natural OW. We validated the performance of the proposed system through mimicking BWSOWT experiments with eight healthy subjects.

## 2. The proposed treadmill training system

The proposed treadmill training system consists of an ITM for simulating OW, an optimized BWS for ITM to provide partial weight bearing, and a harness for connecting the user to the BWS.

### 2.1. Interactive treadmill

Previously, we had developed the ITM, an improved self-paced treadmill (Kim et al., 2014, 2015). The advantages of ITM compared with the existing self-paced treadmill were (1) better similarity to OW caused by its minimal anomalous (inertial) force due to attenuated treadmill belt acceleration (Kim et al., 2014), and (2) closer relevance to clinical use, due to its compact size and low-cost components (Kim et al., 2015). Moreover, we showed that the user's attention to gait on ITM is significantly increased, because the former advantage enables ITM to naturally use a more interactive OW protocol (Oh et al., 2018). Therefore, we developed the BWSOWT system based on our ITM.

**Figure 1:**
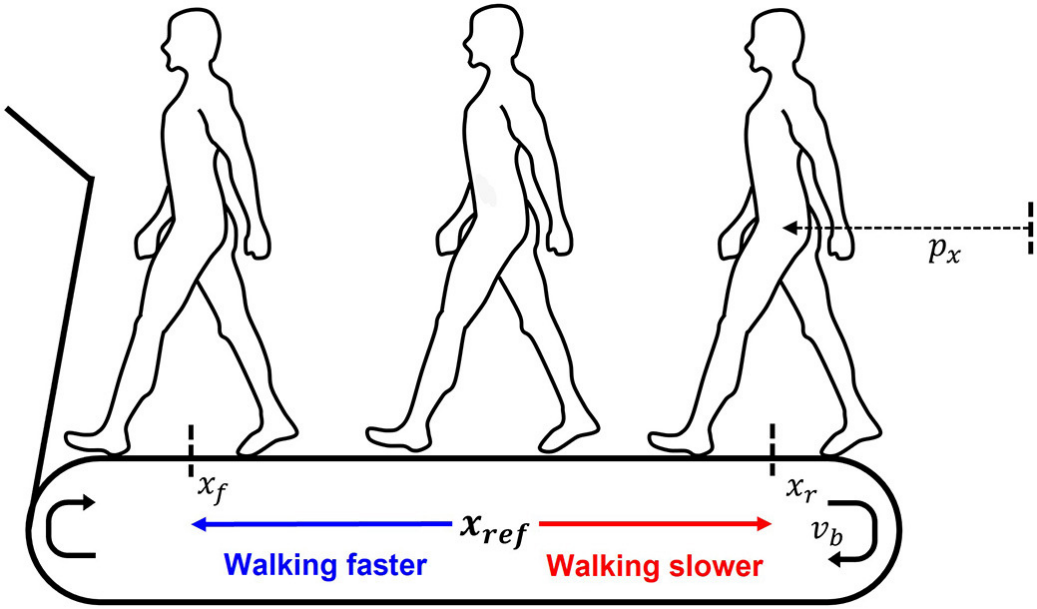
Variable reference according to the user's position (*p*_*x*_; the user's A/P position, *v*_*b*_; a treadmill belt speed, *x*_*f*_; the forefront of the treadmill walking range, *x*_*r*_; the tail position of the treadmill walking range, *x*_*ref*_; the reference position).

Most self-paced treadmills were implemented by treadmill belt speed controllers, whose role is to locate the user at the reference position (middle of the treadmill belt), regardless of his/her walking speed (Minetti et al., 2003; Fung et al., 2006; Feasel et al., 2011). Hence, more belt acceleration is required to guarantee that the user stays within the treadmill with a shorter belt length. The ITM also has a belt speed controller, but the controller has a key characteristic to achieving the advantages of the ITM above; this is to simulate a virtual long-belt treadmill by adjusting the user's reference position of the controller according to the walking speed, as follows (Figure 1) (Souman et al., 2010).


(1) $$\begin{array}{l} x_{ref}=\frac{x_{f}-x_{r}}{V_{w,max}}{Ṽ}_{w}\left(v_{b},\;p_{x}\right)+x_{r}, \end{array}$$

Where, *x*_*f*_ and *x*_*r*_ denote the forefront and the tail position of the treadmill walking range in the anterior/ posterior (A/P) direction, respectively; Ṽ_*w*_ the user's walking speed estimated by using the speed of treadmill belt (*v*_*b*_) and the user's position in the A/P direction (*p*_*x*_); and *V*_*w,max*_ the maximum allowable walking speed. It was shown that the required belt acceleration for simulating OW, which directly affects anomalous force, can be significantly reduced due to the virtual belt lengthening effect through Eq. (1) (Souman et al., 2010). Instead, the user's position on the ITM is substantially changed according to the user's walking speed (Figure 1). Note that ITM needed to acquire the user's position like other self-paced treadmills, and in our previous studies (Kim et al., 2014, 2015; Oh et al., 2018), motion capture system or motion sensor were used to measure the position.

### 2.2. Two wire-driven body weight support mechanism

**Figure 2:**
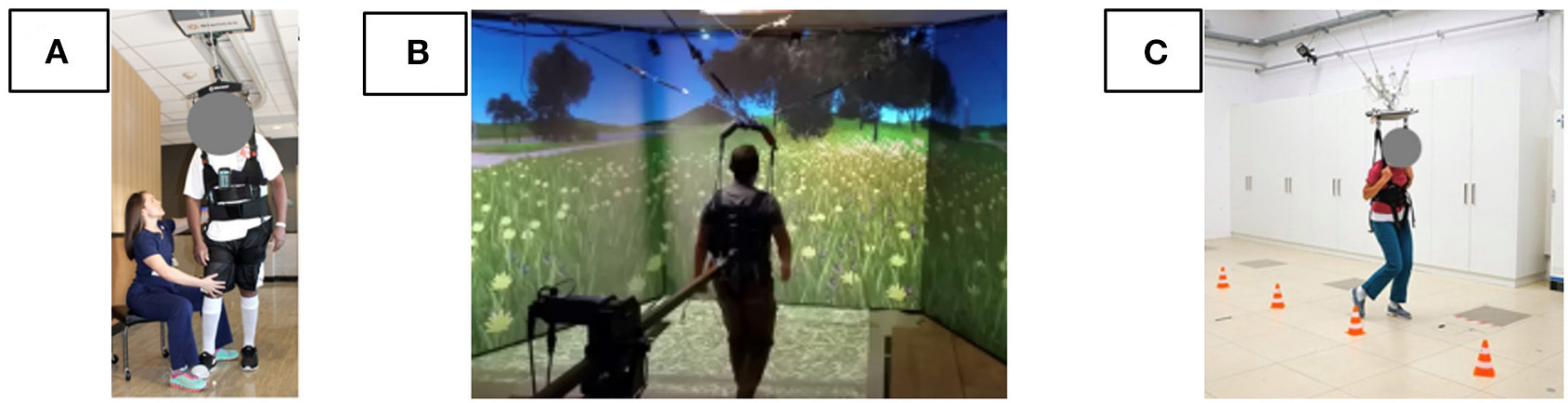
Conventional BWSOWT **(A)** a single wire, **(B)** three wires, and **(C)** four wires.

The conventional BWS to partially offload the user's weight generally used a single wire (Hidler et al., 2011), even for BWSOWT (Hidler et al., 2011), as displayed in Figure 2A. Since the unloading force generated by this method is very sensitive to the direction of the wire, several studies attempted to use three wires (Sabetian and Hollerbach, 2017), or four wires (Vallery et al., 2013) to improve the quality of unloading force (Figures 2B, C). However, those methods required large workspace and high cost, so are not appropriate to the approach to BWSOWT.

**Figure 3:**
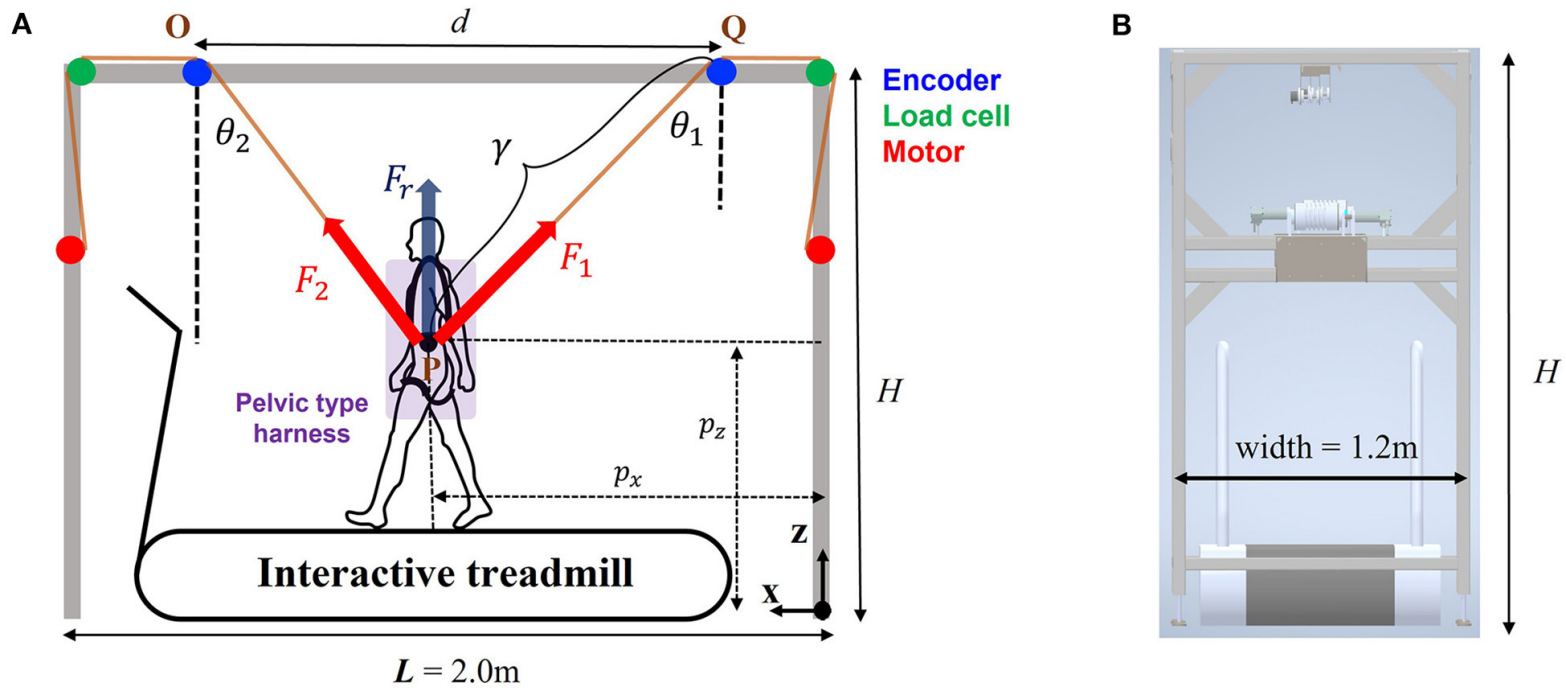
Schematic diagram of the proposed system. **(A)** Frontal view, and **(B)** side view (L; length of the system, H; height of the system, θ_1_ and θ_2_; wire angle, *l*_1_ and *l*_2_; wire length, d; encoder distance, *F*_1_; desired tension of wire 1, *F*_2_; desired tension of wire 2, *F*_*r*_; desired unloading force).

Thanks to ITM, we were able to implement OW in a small workspace. To keep this merit, we needed to simplify the structure of the BWS. On the other hand, ITM allows the user significant A/P movement on treadmill, so we needed to consider the effect of this movement on the unloading force. Therefore, with the assumption that treadmill walking can be approximated on sagittal walking, a novel two wire-driven mechanism was proposed as an optimal design for BWSOWT based on ITM, as illustrated in Figure 3. To provide support force for partial weight bearing only, the following conditions must be satisfied:


(2) $$\begin{array}{l} \sum F_{x}=F_{2}\sin\theta_{2}-F_{1}\sin\theta_{1}=0;\;\\ \sum F_{z}=F_{2}\cos\theta_{2}+F_{1}\cos\theta_{1}=F_{r}, \end{array}$$

Where, *F*_*x*_ and *F*_*z*_ denote the forces in the A/P and gravitational directions, respectively; *F*_1_ and *F*_2_ the tension forces for left and right wires, respectively; θ_1_ and θ_2_ the angles of each wire; and *F*_*r*_ the reference unloading force. The desired tension forces to meet Eq. (2) for BWS were calculated as follow:


(3) $$\begin{array}{l} F_{1,\;d}=\frac{F_{r}\sin\theta_{2}}{\sin\left(\theta_{1}+\theta_{2}\right)};\;F_{2,d}=\frac{F_{r}\sin\theta_{1}}{\sin\left(\theta_{1}+\theta_{2}\right)}. \end{array}$$

To track the desired tension forces [Eq. (3)], we applied a conventional PI controller with feedforward term, as follows:


(4) $$\begin{array}{l} \begin{array}{l} u_{i}=F_{i,\;d}+\left(K_{p}F_{i,\;e}+K_{I}\int F_{i,\;e}dt\right) \end{array} \end{array}$$

Where, *u*_*i*_ (*i* = 1, 2) denotes the control input for each wire; *F*_*i,d*_ the desired tension force in Eq. (3); *F*_*i,e*_ the force error between the desired and the measured force; and *K*_*p*_ and *K*_*I*_ are the proportional and the integral gain, respectively.

Along with body weight support, another function of the wire mechanism is to measure the user's position. As mentioned in Eq. (1), ITM needs the user's position (P) in the A/P direction (*p*_*x*_), so additional sensors were used to measure it (Kim et al., 2014, 2015). On the other hand, this system enabled the user's position to be obtained without any sensor, as follows (Figure 3).


(5) $$\begin{array}{l} p_{x}=\frac{\left(L-d\right)}{2}+\;\gamma \sin\theta_{1};\;p_{z}=H-\gamma \cos\theta_{1}, \end{array}$$

Where, *p*_*z*_ denotes the user's position in the cranial/caudal direction; *d* the encoder distance (OQ); γ the wire length that was determined by the law of sines for triangle OPQ; θ_1_ the wire angle; and *L* and *H* the length and the height of the proposed system, respectively.

### 2.3. System design

For the proposed treadmill-based mimicking BWSOWT system, a speed controllable treadmill needs to be considered to implement the ITM. Based on a well-known controllable treadmill (Woodway PPS Med 55, USA), we chose the design parameters, *L* and width, of the two-wire driven BWS as 2.0 and 1.2 m, respectively (Figure 3).

Due to the geometry (triangle OPQ) of the BWS in Figure 3, the parameter *d* can change θ_1_ and θ_2_ with the same P (user's position), and the parameter *H* could affect θ_1_ and θ_2_ with the same user (height). Thus, the parameters *d* and *H* determine the required tension forces in Eq. (3), which is closely related to the actuator specification of the system. Moreover, *H* determines the required space of the system. Therefore, we conducted a simulation to choose *d* and *H* for a cost-effective and compact system for clinical use. For this simulation, we virtually generated a user's trajectory (*p*_*x*_ and *p*_*z*_) that mimics an actual walking training, and obtained the required tension of each wire using Eq. (3) with calculated θ_1_ and θ_2_ under various *d* and *H*, as follows:


(6) $$\begin{array}{l} \theta_{1}=tan^{-1}\;(\frac{d-\beta }{\alpha });\;\theta_{2}=tan^{-1}\left(\frac{\beta }{\alpha }\right),\\ \;with\;\alpha =H-p_{z};\beta =\frac{L+d}{2}-p_{x} \end{array}$$

**Figure 4:**
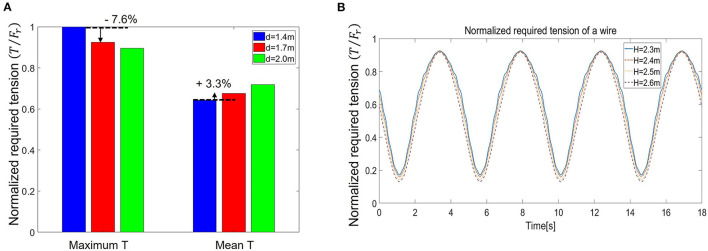
Simulation results of required tension **(A)** Normalized maximum and mean required tension, and **(B)** required a wire tension with various system heights (H).

Here, the walking range for the trajectory was set to *p*_*x*_ length 1.4 m (about 91.5 % of the walkable range of the Woodway treadmill), and *p*_*z*_ amplitude 0.03 m (Hidler et al., 2011). As displayed in Figure 4, the simulation result showed that (1) choosing *d* is a trade-off between reducing the maximum required tension and increasing the mean required tension, and (2) the effect of *H* is not significant. Therefore, we chose *d* as 1.7 m as a moderate solution, and *H* as 2.3 m to reduce the required space of the system.

### 2.4. Pelvic-type harness

**Figure 5:**
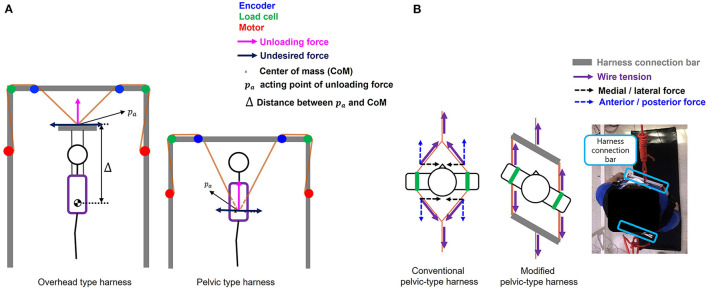
Comparison between **(A)** Overhead type harness and pelvic type harness, and **(B)** conventional pelvic type harness and modified pelvic type harness.

Most BWSs have used an overhead-type harness to provide unloading force (Koenig et al., 2009; Hidler et al., 2011; Vallery et al., 2013; Sabetian and Hollerbach, 2017), but we opted for pelvic-type harness (Figure 5A), because it is beneficial (1) to reduce the height (volume) of our BWS, and (2) to relieve the pendulum effect, due to its short distance between the acting point of the unloading force and the user's center of mass (Wyss et al., 2014).

Despite those benefits, the conventional pelvic-type harness has a potential drawback from the viewpoint of pelvic motion during BWSOWT. Since wire tension is directly applied to the user's pelvis under the harness, it would significantly deteriorate natural pelvic motion, which affects core learning in BWSOWT: gait pattern and gait balance (Jung et al., 2014). Therefore, we modified the design of the conventional pelvic-type harness by adding connection bars, as shown in Figure 5B. Thanks to the bars, the harness enabled free pelvic movement by elimination of the M/L wire tension (Figure 5B).

### 2.5. System implementation

**Figure 6:**
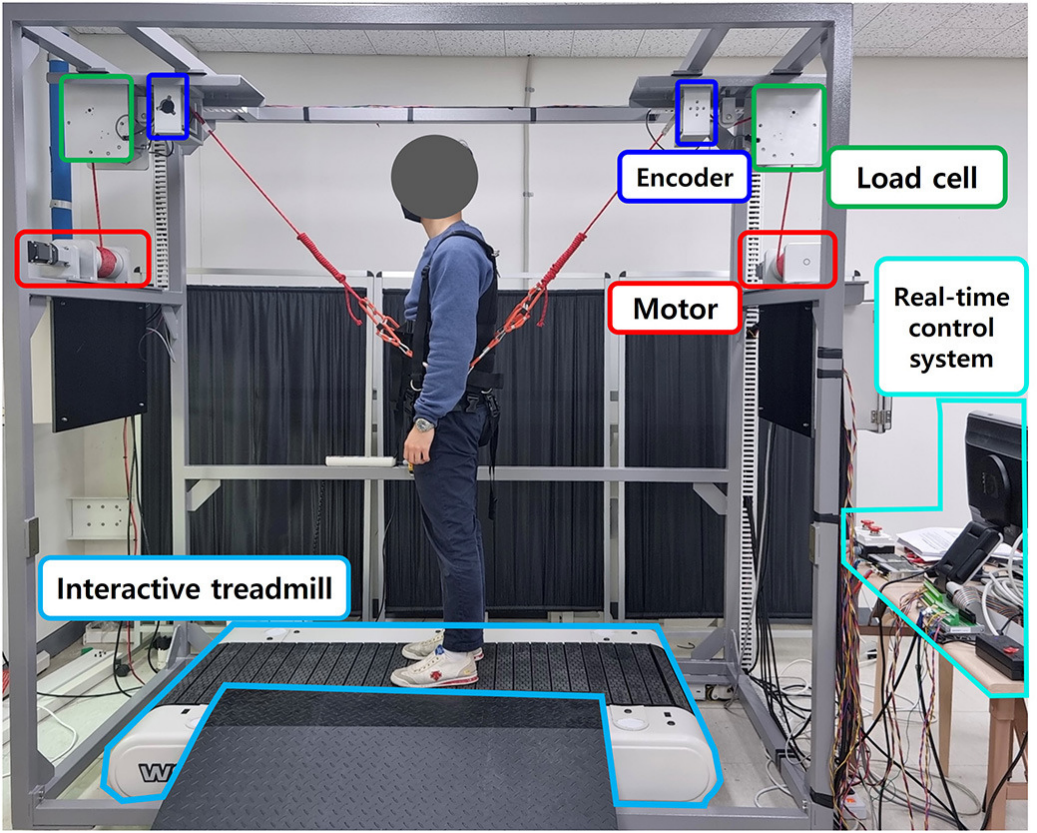
The proposed BWSOWT system.

Based on the ITM, the two-wire driven BWS, and the pelvic-type harness, we implemented a proposed mimicking BWSOWT system based on the treadmill, as shown in Figure 6. As mentioned, we adopted a speed-controllable treadmill (Woodway PPS Med 55) for ITM, and the BWS was used to provide unloading force and to measure the user's position for ITM. Through the custom pelvic-type harness, the user was connected to the proposed system for more natural pelvic movement.

The ITM was connected to a single board computer (PCM-3365EW, Advantech, Taiwan) *via* USB for RS-232 serial communication and communicated with a 30 Hz sampling frequency for the belt speed controller. The BWS used two DC motors (422,969, Maxon motor, Switzerland) to generate the unloading force, and the included load cells and rotary encoders measured the wire tensions and wire angles, respectively. The motor drivers (Maxon motor, Switzerland), the loadcells (Transducer techniques, USA), and the rotary encoders (E40S6-5000-6-L-5, Autonics, Korea) in the BWS were connected to a data acquisition board (Model 526, Sensoray, USA) installed in the computer. The force controller [Eq. (3)] for the BWS was implemented by C++ under a real-time operating system (Xenomai) with a 1 kHz sampling frequency.

## 3. Experiments

### 3.1. Experimental setup

To evaluate the proposed system experimentally, we conducted a body weight support overground walking training (BWSOWT) using the developed system. In the experiments, we focused on evaluating the following objectives of the proposed system: (1) mimicking OW of ITM, (2) providing unloading force of BWS, and (3) allowing natural pelvic motion during the training. Since it was already verified through several existing works that the ITM's performance on simulating OW is affordable with valid A/P position (*p*_*x*_) of the user (Souman et al., 2010; Kim et al., 2014, 2015), we compared the position obtained from the BWS by Eq. (4) with that measured by a motion capture system (VICON nexus, UK). Moreover, both the error of unloading force and the amount of pelvic motion were calculated during the BWSOWT. Note that all gains in the force controller [Eq. (4)] were manually tuned, and the maximum walking speed was limited to 1.6 m/s for safety.

With considering the number of participants in the pilot studies of the existing systems (Hidler et al., 2011; Vallery et al., 2013; Sabetian and Hollerbach, 2017), we recruited eight healthy subjects (5 males, 3 females) for the experiments. The experiments were approved by the institutional review board (SKKU 2022-06-014), and all participants were given the details of the experiment.

### 3.2. Protocols and data analysis

**Figure 7:**
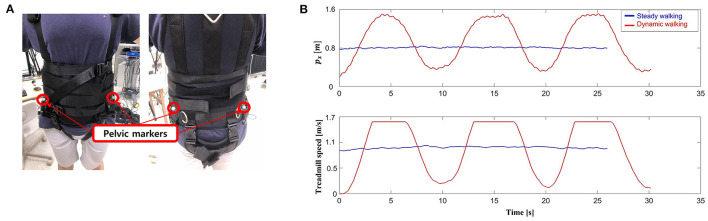
Experiment setup. **(A)** Marker attachment, and **(B)** experimental tasks.

We first conducted a pre-experiment session to measure the reference pelvic position, to find the subjects' preferred walking speed, and to provide practice before the experiment. The subjects were asked to wear the harness and attach four passive markers on left/right ASIS and left/right PSIS (Figure 7A). They maintained the static standing posture on the treadmill to capture the position of each marker, and walked on the treadmill with 120 N unloading force for (2–3) min to find their preferred speed, and to become accustomed to the system.

After the session, we conducted the mimicking BWSOWT experiments with both conditions: maintaining subjects' preferred walking speed (steady walking), and changing the walking speed that results in forward/backward movement within the treadmill (dynamic walking) (Figure 7B). This is because: (1) we want to fairly compare our force control performance with the existing BWSOWT systems which are only available for steady walking, and (2) we also want to evaluate the performance with dynamic walking, which is the characteristics of ITM. The subjects were asked to walk 20 strides for each condition with five reference unloading forces of (60, 120, 180, 240, and 300 N). Note that the reference unloading forces were set as absolute value to compare its force control performance with existing studies. First, for steady walking, they started to walk at the tail position of the treadmill walking range (*x*_*r*_), and walked 20 strides with their preferred walking speed. After that, they rested for (3–5) min, and then engaged in dynamic walking. For dynamic walking, they walked 10 strides with slow speed (80 % of their preferred speed), and 10 strides with fast speed (120 % of their preferred speed) (Oh et al., 2018).

**Figure 8:**
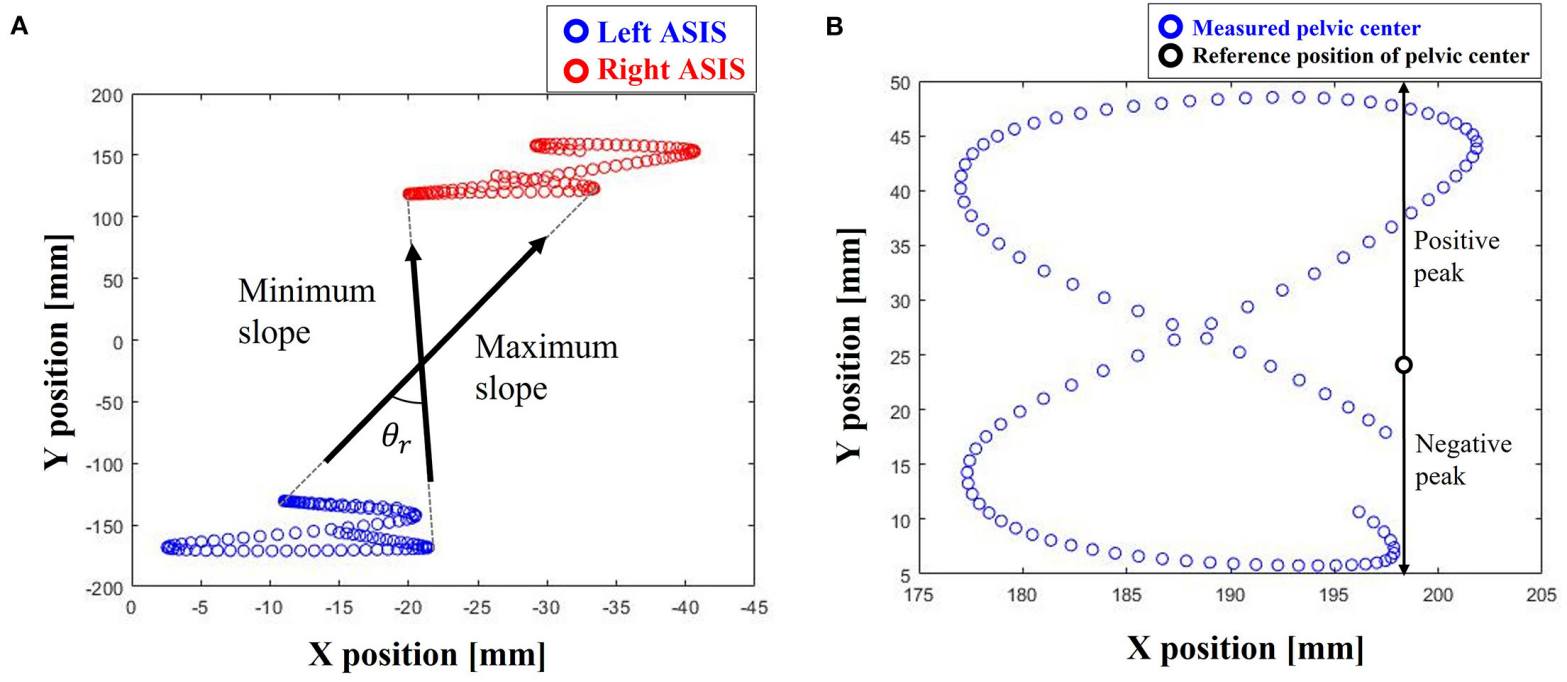
Definition of pelvic motions. **(A)** Rotation (θ_*r*_; rotation angle), and **(B)** translation (difference between positive peak and negative peak).

We calculated the root mean square error (RMSE) between the user's A/P position obtained by Eq. (4) and the A/P axis position of the user's pelvic center calculated by the marker positions with the motion capture system (Stokes et al., 1989). The RMSE between the actual unloading force obtained by Eq. (2) and the reference unloading force was calculated to evaluate the unloading force. Moreover, we measured the undesired A/P directional force that could appear due to the structural characteristics of the 2-wire BWS. The pelvic motion can be classified as pelvic rotation and translation (Stokes et al., 1989). The amount of pelvic rotation was calculated as the angle between the line's maximum slope and the minimum slope connecting the left ASIS marker and right ASIS marker, while the pelvic translation was calculated from positive peak to negative peak, based on the pelvic center (Figure 8) (Stokes et al., 1989).

We hypothesized that position error and pelvic motion were no significant differences among unloading force levels and walking conditions, respectively. Since the data were not normally distributed (Shapiro-Wilk test, α = 0.05, *p* < 0.01), we conducted the Friedman test among five unloading force levels (Sawilowsky and Fahoome, 2005) and the Wilcoxon signed rank test between two walking conditions (Rey and Neuhäuser, 2011) for statistical analysis. Note that the Friedman test and Wilcoxon signed rank test were performed under two-tailed test.

## 4. Results

As mentioned, the subjects asked to walk with two conditions: maintaining their preferred walking speed (steady walking) and changing their walking speed (dynamic walking).

### 4.1. Position sensing for interactive treadmill

**Figure 9:**
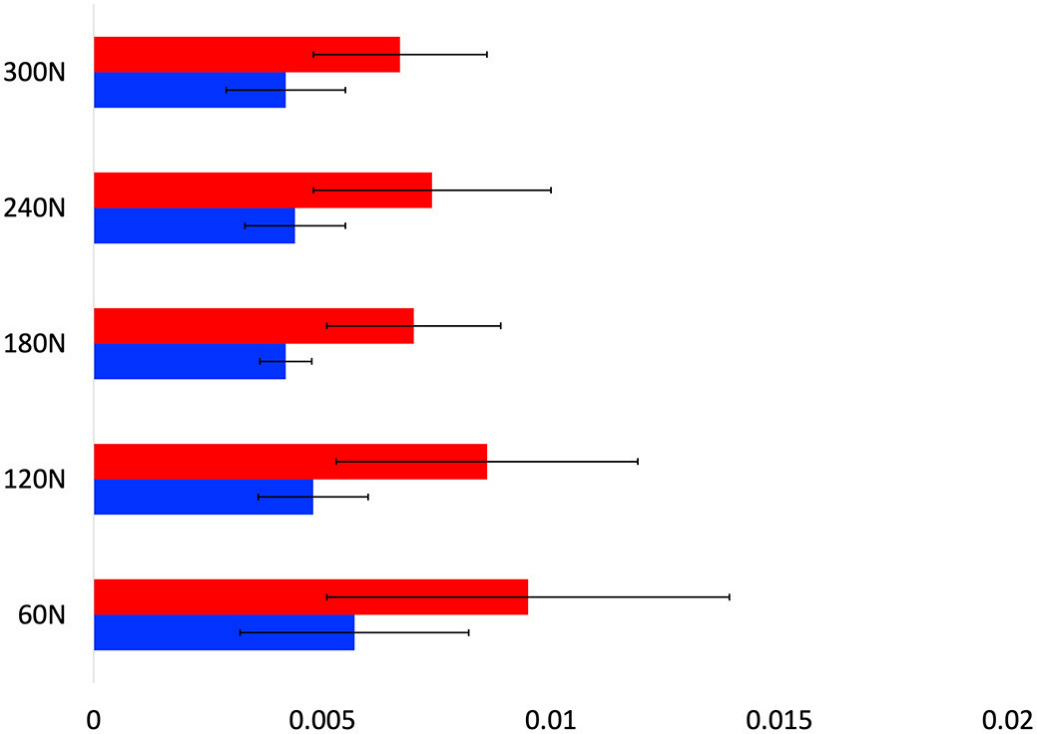
User's A/P position error [Red bar; dynamic walking, blue bar; steady walking, Green dotted line: reference value in Khoshelham and Elberink (2012)].

For the performance on the ITM, we evaluated the accuracy of the subject's A/P position obtained by the proposed system. Table 1 and Figure 9 show the position errors between the proposed system and the motion capture system. Compared with the measurement error of the motion sensor (up to 13 mm) used for ITM previously (Khoshelham and Elberink, 2012), the proposed system can provide more accurate position (Figure 9). This result implies that the ITM performance, simulating OW, of the proposed system is at least comparable to the previous ITM (Kim et al., 2014, 2015). As shown in Table 1, there was no significance in the subject's A/P position error differences among the unloading force levels and walking conditions (0.081 ~ 0.157, minimum ~ maximum of *p*-value). It means that various mimicking BWSOWT conditions, such as the level of unloading force and/or steady/dynamic walking, using the proposed system are all appropriate to mimic overground walking.

**Table 1:** User's A/P position error and statistical results with 95% confidence interval (CI).

**Unloading force**	**Position error [mm]**			
	**Steady walking (mean** ± **SD)**	**Dynamic walking (mean** ± **SD)**	**Mean difference (95% CI lower bound, 95% CI upper bound)**	**Wilcoxon signed rank test (** ***p*** **-value)**
60 N	5.7 ± 2.5	9.5 ± 4.4	−0.15 (−0.41, 0.10)	0.157
120 N	4.8 ± 1.2	8.6 ± 3.3	−0.55 (−0.77, −0.33)	0.081
180 N	4.2 ± 0.57	7 ± 1.9	−3.21 (−5.91, −2.53)	0.095
240 N	4.4 ± 1.1	7.4 ± 2.6	−0.35 (−0.56, −0.13)	0.128
300 N	4.2 ± 1.3	6.7 ± 1.9	−1.55 (−2.22, −0.84)	0.093
Friedman test (*p*-value)	0.112	0.122		

### 4.2. Unloading force generation

Table 2 shows the performance of the unloading force of the proposed system under the two walking conditions. For the unloading force for supporting body weight, the proposed system showed comparable performance; the amount of unloading force RMSE of the proposed system was similar to the existing BWSOWT systems [FLOAT: 7.2–9.6 (Vallery et al., 2013), 3-wire BWS: 2.6–5.0 (Sabetian and Hollerbach, 2017), and Zero-G: 3.0-3.9 (Hidler et al., 2011)]. Furthermore, the proposed system caused much lower undesired force than the existing BWSOWT systems [FLOAT: 6.6–9.7 (Vallery et al., 2013) and 3-wire BWS: 4.0–8.4 (Sabetian and Hollerbach, 2017)] (Table 1). Overall, the results show that despite the simplicity and small required space of the proposed system, the quality of unloading force provided by the proposed system is comparable to that of the existing BWSOWT system.

**Table 2:** Control performance of the proposed system.

**Unloading force**	**Unloading force RMSE [** ***N*** **]**		**Undesired force RMSE in AP direction [N]**	
	**Steady walking (mean** ± **SD)**	**Dynamic walking (mean** ± **SD)**	**Steady Walking (mean** ± **SD)**	**Dynamic walking (mean** ± **SD)**
60 N	5.9 ± 2.2	7.1 ± 3.2	1.4 ± 0.3	1.8 ± 0.6
120 N	5.8 ± 1.9	7.3 ± 3.1	1.2 ± 0.3	2.4 ± 1.2
180 N	6.7 ± 2.1	8.4 ± 3.2	1.3 ± 0.4	1.9 ± 0.8
240 N	7.2 ± 2.7	8.3 ± 2.8	1.4 ± 0.6	1.8 ± 0.6
300 N	8.7 ± 2.6	10.6 ± 3.2	1.4 ± 0.7	2.3 ± 0.9

### 4.3. Pelvic motion

**Figure 10:**
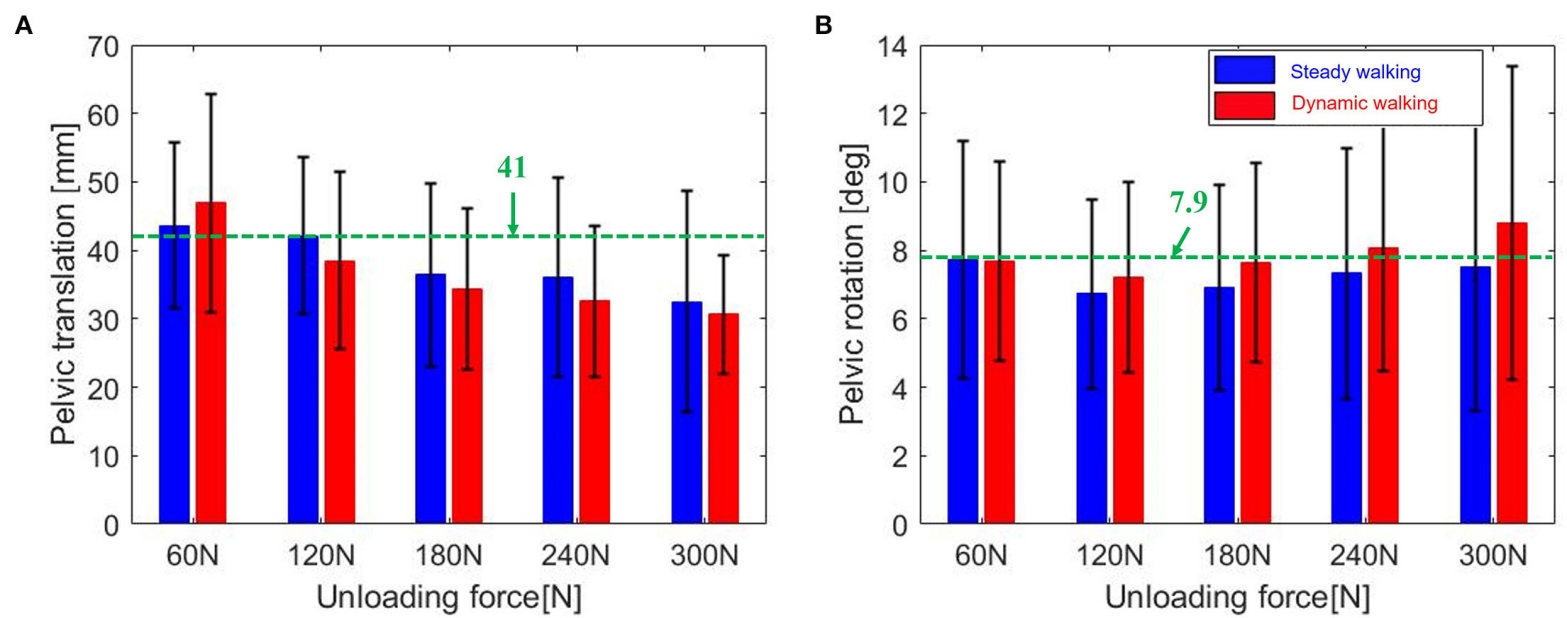
**(A)** Pelvic translation, and **(B)** rotation [Red bar; dynamic walking, blue bar; steady walking, Green dotted line: reference value in Stokes et al. (1989)].

BWS is to help the learning of gait pattern, and pelvic motion has large effects on the learning of gait pattern (Jung et al., 2014). Hence, we checked the pelvic motion during mimicking BWSOWT. Table 3 and Figure 10 show the amount of pelvic motion during the walking with the proposed system, and that the proposed system can allow enough pelvic motion, which is similar to the previous work that used a BWS system with overhead-type harness pelvic translation: (4.1 ± 1.5) cm and pelvic rotation: (7.9 ± 1.5) deg (Stokes et al., 1989) (Figure 10). As shown in Table 3, there were no significant pelvic motion differences among the unloading force levels and walking conditions (0.074 ~ 0.813, minimum ~ maximum of *p-*value). This implies that various mimicking BWSOWT conditions, such as the level of unloading force and/or steady/dynamic walking, using the proposed system are all appropriate for the learning of gait pattern.

**Table 3:** Pelvic motion and statistical results with 95% confidence interval (CI).

**Unloading force**	**Translation [mm]**				**Rotation [deg]**			
	**Steady walking (mean** ± **SD)**	**Dynamic walking (mean** ± **SD)**	**Mean difference (95% CI lower bound, 95%CI upper bound)**	**Wilcoxon signed rank test (** ***p*** **-value)**	**Steady walking (mean** ± **SD)**	**Dynamic walking (mean** ± **SD)**	**Mean difference (95% CI lower bound, 95%CI upper bound)**	**Wilcoxon signed rank test (** ***p*** **-value)**
60 N	43.6 ± 12.1	46.9 ± 16.0	3.12 (−1.51, 7.59)	0.157	7 ± 3.3	6.8 ± 3.3	0.10 (−0.71, 0.78)	0.813
120 N	42.1 ± 11.5	38.5 ± 12.9	1.94 (−3.54, 7.43)	0.480	5.4 ± 2.3	6.3 ± 3.1	−0.11 (−0.28, 0.17)	0.157
180 N	36.4 ± 13.4	34.4 ± 11.7	2.21 (−1.33, 6.14)	0.157	4.4 ± 2.3	6.3 ± 2.8	−0.16 (−1.04, 0.31)	0.480
240 N	36 ± 14.6	32.6 ± 10.9	1.56 (−0.09, 3.21)	0.074	5.8 ± 2.9	6.3 ± 3.2	−0.18 (−0.41, 0.04)	0.157
300 N	32.5 ± 16.2	30.7 ± 8.7	2.18 (−0.79, 5.35)	0.157	5.4 ± 2.8	6.1 ± 3.2	−0.17 (−0.65, 0.21)	0.157
Friedman test (*p*-value)	0.717	0.791			0.518	0.645		

## 5. Discussion

The goal of this study is to develop a cost-effective and non-oversized treadmill-based gait training system to mimic natural OW with body weight support. For that, we opted for the interactive treadmill that can simulate overground walking within a small space. The BWS was designed based on a two-wire mechanism with considering the characteristic of the treadmill, to be as compact as possible. Moreover, we developed the custom pelvic-type harness that allows natural pelvic motion during gait. Through the experiments with healthy subjects, we verified that the proposed system could enable BWSOWT while providing affordable unloading force (weight bearing). Furthermore, we expect that the proposed system could facilitate better brain plasticity during BWSOWT, due to the attentional advantage of the interactive treadmill verified in (Oh et al., 2018).

As regards the space-effectiveness of the proposed system, Table 2 summarizes the comparison of the required volume between the proposed system and the existing BWSOWT systems. Thanks to its compact design, the proposed system requires the smallest space (Table 4). On the other hand, the proposed system cannot implement turning and medial/lateral directional movements.

**Table 4:** Dimension of the proposed system and existed systems.

**Zero-G**	** 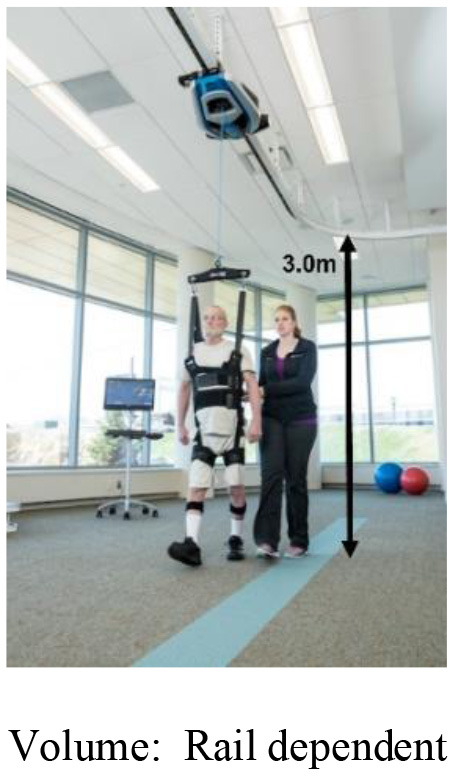 **	**FLOAT**	** 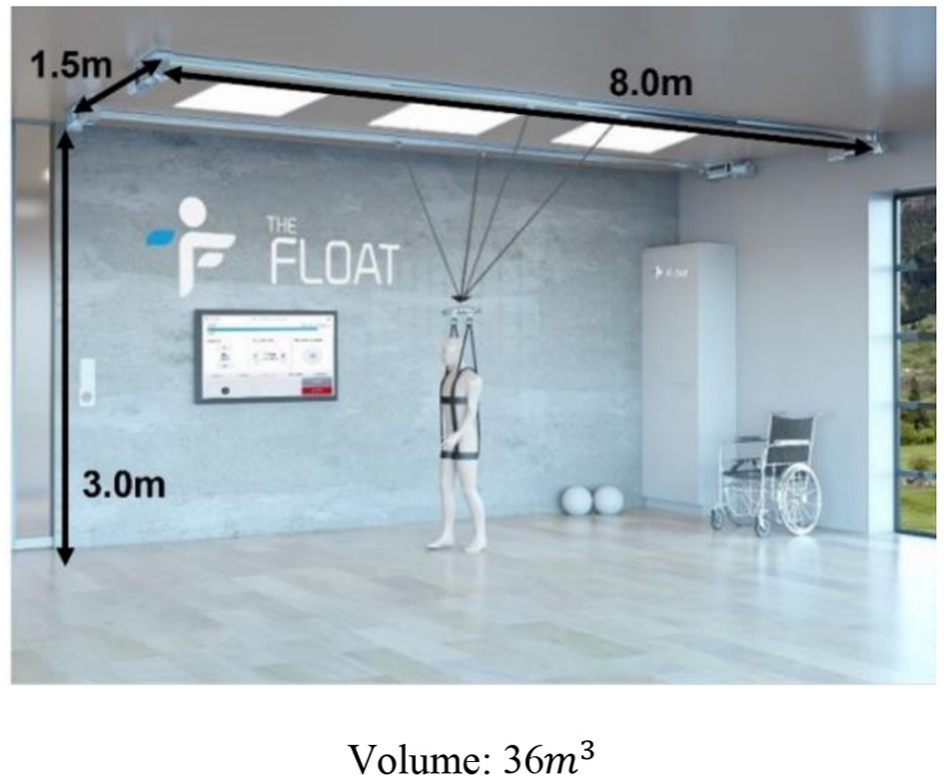 **
3 wire BWS	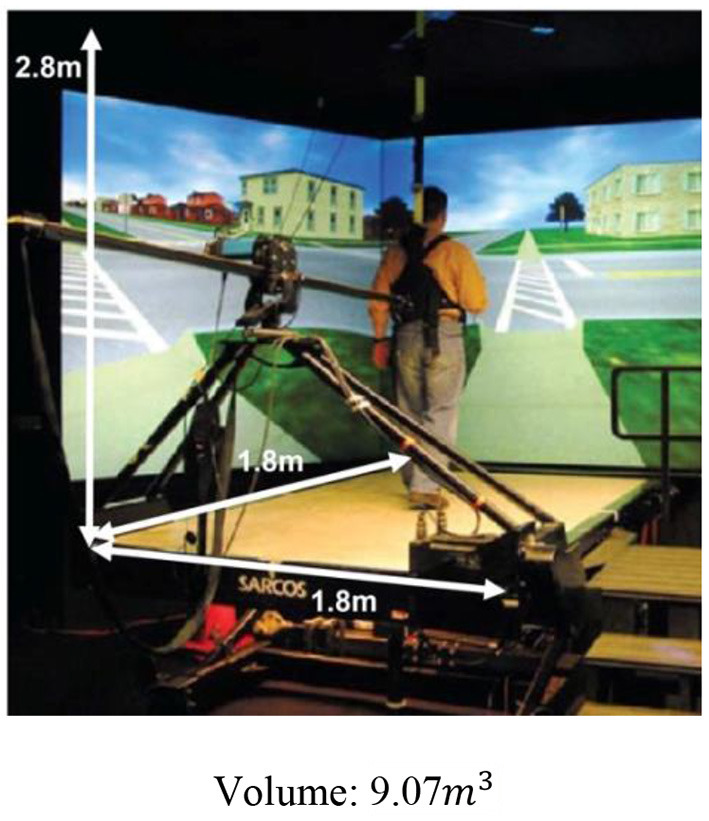	The proposed system	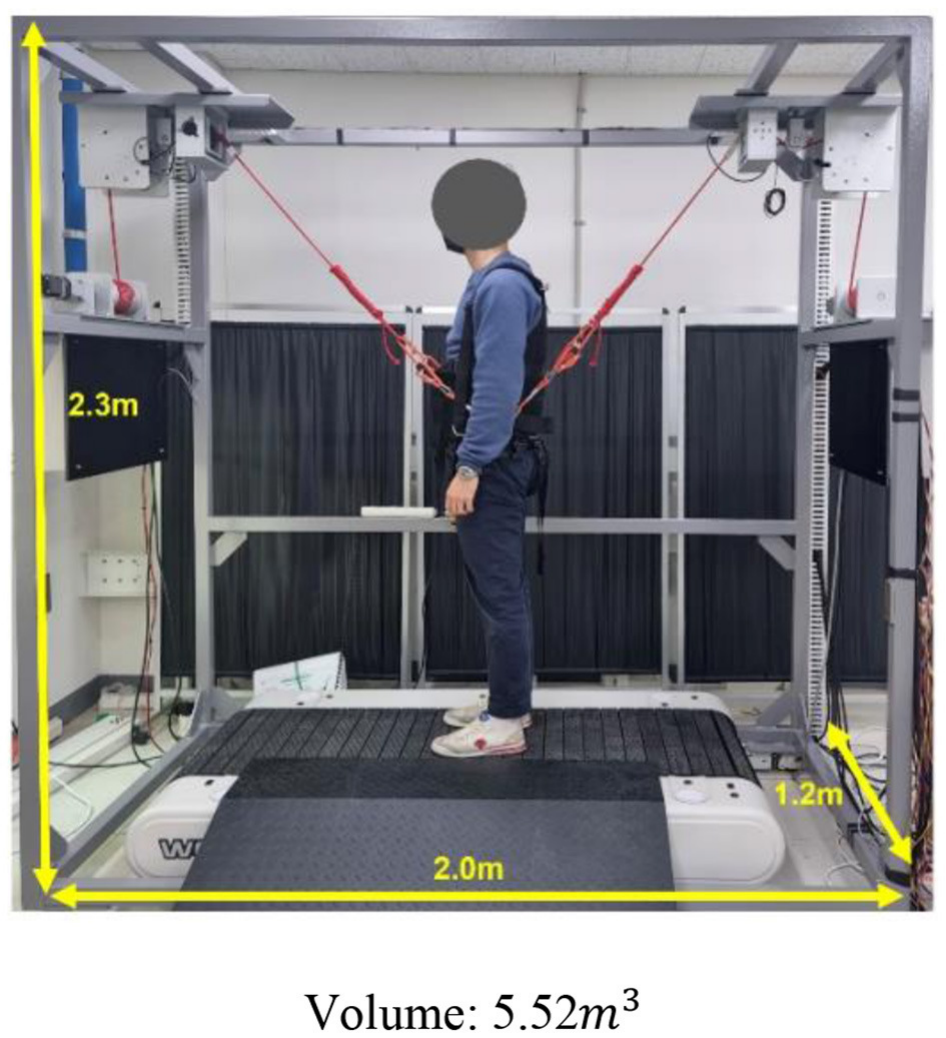

As mentioned, the proposed system showed comparable unloading force control performance, but the performance of the existing systems Zero-G and 3-wire was slightly better than that of the proposed system. This result would come from the following reasons. First, the proposed system used a conventional actuator (DC motor) to provide unloading force, while those existing systems used a high-grade and expensive actuator (series elastic actuator). It is well-known that the series elastic actuator offers superior force control (Vallery et al., 2008). Another difference of our study is that the subject's walking speed during the experiment of (0.8 ± 0.04 m/s) was quite faster than the walking speed in the existing studies of (0.45 ± 0.03 m/s) (Hidler et al., 2011; Vallery et al., 2013). In general, faster walking speed would cause larger unloading force error, due to greater change of pelvic position [*p*_*z*_ in Eq. (4)] in the cranial/caudal direction (Stokes et al., 1989). It should be noted that the proposed system showed better performance on the level of undesired force than Zero-G and 3-wire, which could directly affect gait pattern.

Despite its meaningful start, the proposed system still has room for improvement. First, we need to investigate the similarity between actual OW and mimicked OW with and the proposed system with respect to joint kinematics/kinetics and EMG activities. As we used a 2-wire mechanism based BWS for simplicity, the force in the medial/lateral direction could not be controlled. Although this effect was negligible in the BWSOWT trials with healthy subjects, we have to investigate the significance of the effect with a greater population, including patients with impaired gait. The control performance also needs to be optimized to provide more accurate unloading force. Moreover, an optimized protocol for the proposed system needed to be investigated for future clinical use (Oh et al., 2022).

## Data availability statement

The distribution of raw data supporting the conclusions of this article is strictly controlled by the IRB and prohibited for privacy protection. Further inquiries can be directed to the corresponding author/s.

## Ethics statement

The studies involving human participants were reviewed and approved by the Institutional Review Board of Sungkyunkwan University (SKKU 2022-06-014). The patients/participants provided their written informed consent to participate in this study.

## Author contributions

JonghK supervised the study, conceptualized and designed the study, acquired the funding, provided the resources for the study, and finalized the manuscript. JongbK implemented the proposed system and drafted the original manuscript. JongbK and SO designed the experiments. JongbK, SO, YJ, and JM recruited subjects, prepared IRB for the experiments, and interpreted results from the data. All authors read and revised the manuscript and approved the final manuscript for publication.
